# Video-assisted thoracoscopic surgery involving a bronchotomy in the removal of a bronchial foreign body: A novel case report

**DOI:** 10.1016/j.ijscr.2024.110018

**Published:** 2024-07-09

**Authors:** Fumiya Kawano, Mayu Inomata, Ryusei Yamada, Ryo Maeda

**Affiliations:** Department of Thoracic and Breast Surgery, Faculty of Medicine, University of Miyazaki, Miyazaki, Japan

**Keywords:** Bronchotomy, Bronchial foreign body, Chest roentgenography, Medical emergency, Video-assisted thoracoscopic surgery

## Abstract

**Introduction and importance:**

Bronchial foreign body aspiration is a life-threatening emergency. Largely, the published literature focuses on the removal of foreign bodies by bronchoscopy, while the surgical removal of endobronchial foreign bodies is rarely reported on. Thus, we presented a case of a bronchial foreign body that was successfully removed by a video-assisted thoracoscopic surgical (VATS) bronchotomy, after multiple failed bronchoscopic attempts.

**Case presentation:**

A 77-year-old male patient presented with a 2-month duration of a persistent cough and low-grade fever after undergoing dental treatment. Bronchoscopy revealed a dental crown surrounded by granulation tissue in the right basal bronchus. The patient was referred to our department for open surgery after undergoing multiple unsuccessful extractions. The bronchial foreign body was removed by a VATS bronchotomy. The postoperative course was uneventful, and the patient was discharged 2 days postoperatively without any complications.

**Clinical discussion:**

Most aspirated tracheobronchial foreign bodies can be removed through bronchoscopy; nonetheless, certain aspirated foreign bodies may require surgical intervention. Furthermore, the indications for bronchotomies encompass the failure to remove the foreign body despite repeated attempts, due to immobility, with or without distal bronchial placement. Thoracoscopy is beneficial in providing superior visualization, with an increased likelihood of post-bronchotomy recovery.

**Conclusion:**

VATS bronchotomy is a safe and effective alternative for the removal of bronchial foreign bodies without sacrificing the functioning of the lung parenchyma.

## Highlights

• We presented a case of a bronchial foreign body that was successfully removed by a video-assisted thoracoscopic surgical bronchotomy.
• Thoracoscopy provides better visualization and increases the likelihood of post-bronchotomy recovery.
• Video-assisted thoracoscopic surgical bronchotomy is a safe and effective alternative for the removal of bronchial foreign bodies without sacrificing the functioning of the lung parenchyma.

## 1 Introduction

Bronchial foreign body aspiration is a life-threatening emergency [1]; nevertheless, most aspirated tracheobronchial foreign bodies can be removed via bronchoscopy [[2], [3]]. In a study conducted by Goyal et al. [2], 207 of the 214 cases (97%) of foreign bodies confirmed by bronchoscopy were successfully extracted. Thus, flexible and rigid bronchoscopy are the gold standard treatment for the removal of foreign body removal from the bronchus [2,[4], [5], [6], [7]]. However, when bronchoscopy fails, patients require open surgery, which occurs in <2% of cases [8].

Largely, the published literature focuses on the removal of foreign bodies by bronchoscopy [2,[4], [5], [6], [7]], while the surgical removal of endobronchial foreign bodies is uncommonly reported on. Consequently, we presented a case of a bronchial foreign body that was successfully removed by a video-assisted thoracoscopic surgical (VATS) bronchotomy, after multiple failed bronchoscopic attempts.

This study was conducted in accordance with the principles of the Declaration of Helsinki and SCARE 2023 guidelines [9].

## 2 Case presentation

**Fig. 1:**
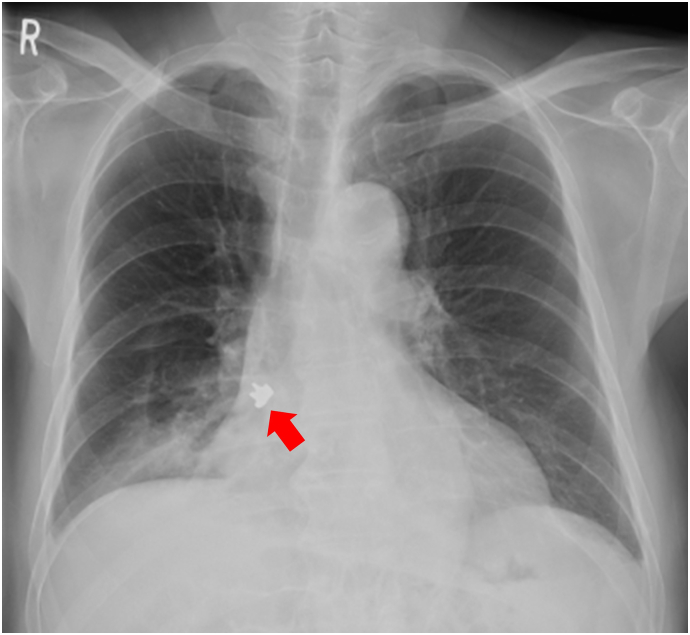
Chest roentgenography revealing a metallic foreign body (red arrow) in the right bronchus. (For interpretation of the references to colour in this figure legend, the reader is referred to the web version of this article.) (For interpretation of the references to colour in this figure legend, the reader is referred to the web version of this article.)

**Fig. 2:**
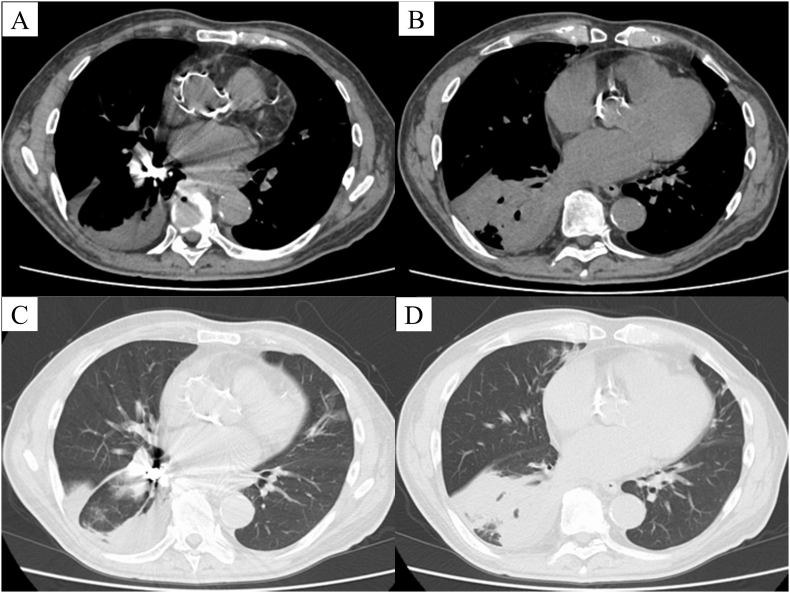
Thoracic computed tomographic (CT) images are shown. (A) Thoracic CT revealing a metallic foreign body, with artifacts in the right basal bronchus; (B–D) in addition to infiltration and consolidation in the right lower lobe and a small pleural effusion.

**Fig. 3:**
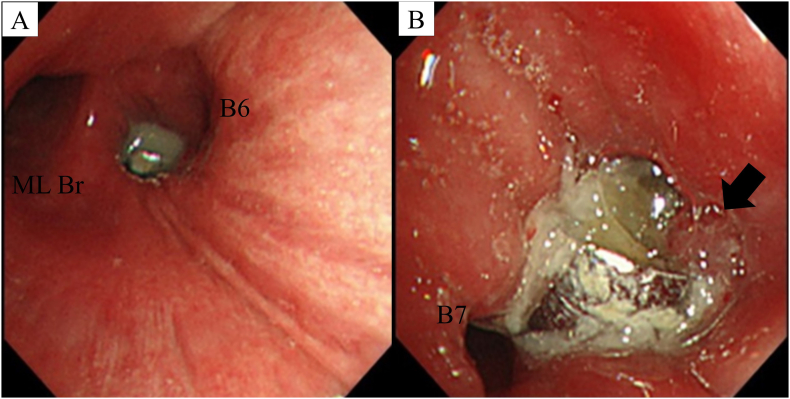
(A–B) Bronchoscopy revealing a dental crown surrounded by a granulation (black arrow) in the right basal bronchus. Abbreviations: ML Br = middle lobe bronchus; B6 = apical basal segment; B7 = anterior basal segment.

A 77-year-old male patient presented with a persistent cough and low-grade fever for 2 months after undergoing dental treatment. Chest roentgenography revealed the presence of a metallic foreign body in the right bronchus (Fig. 1). Computed tomography (CT) revealed a metallic foreign body with artifacts in the right basal bronchus, infiltration and consolidation in the right lower lobe, and a small pleural effusion (Fig. 2A–D). Bronchoscopy showed a dental crown surrounded by granulation in the right basal bronchus (Fig. 3A–B). Flexible bronchoscopic extraction was attempted several times. Nonetheless, the dental crown could not be removed, due to being tightly stuck in the bronchus; in addition to bleeding from granulation tissue. The patient was referred to our department for open surgery after undergoing multiple unsuccessful extractions.

**Fig. 4:**
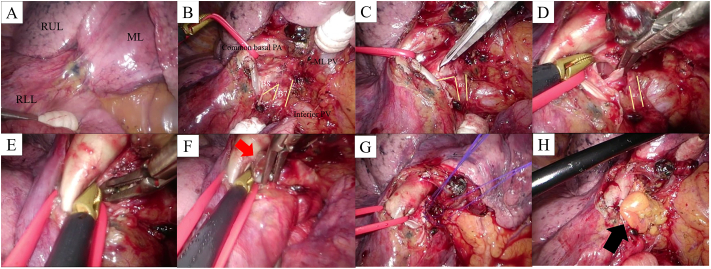
Intraoperative findings are presented. (A–B) The right inferior lobe bronchus has been reached from the anterior aspect of the pulmonary hilum. (C) A longitudinal incision, approximately 1.0 cm-in-length, is made in the anterior cartilage of the right basal bronchus, extending to the right anterior basal segmental bronchus (B8). (D–F) Through this incision, the metallic foreign body (red arrow) is grasped and extracted. (G) The incision is closed with separate 4–0 polypropylene sutures. (H) The bronchial site of the suture is protected by wrapping with a pericardial fat flap (black arrow). Abbreviations: RUL = right upper lobe, ML = middle lobe, RLL = right lower lobe, PA = pulmonary artery, PV = pulmonary vein, B7 = anterior basal segment. (For interpretation of the references to colour in this figure legend, the reader is referred to the web version of this article.) (For interpretation of the references to colour in this figure legend, the reader is referred to the web version of this article.)

Based on the diagnosis of a foreign body in the right basal bronchus and the subsequent complications occurring within the right lung, emergent surgery was performed. During the surgery, the patient was placed in the left lateral decubitus position. Two 1.5 cm working ports were inserted into the fifth intercostal space with non-rib spreading; and a 1 cm camera port was inserted into the seventh intercostal space, at the mid-axillary line. Post-dissection of the adhesion, the right inferior lobe bronchus was reached from the anterior aspect of the pulmonary hilum (Fig. 4A–B). A longitudinal incision, approximately 1.0 cm-in-length, was made in the anterior cartilage of the right basal bronchus, extending to the right anterior basal segmental bronchus (B8) (Fig. 4C). Through this incision, a metallic foreign body, measuring 2 × 1 × 1 cm, was grasped and extracted (Fig. 4D–F). The incision was closed with separate 4–0 polypropylene sutures (Fig. 4G). The right lung immediately re-expanded and appeared to fully ventilate, without air leakage from the repaired bronchial incision. The bronchial site of the sutures was protected by wrapping with a pericardial fat flap (Fig. 4H). The operative time was 85 min, and blood loss was <10 mL.

**Fig. 5:**
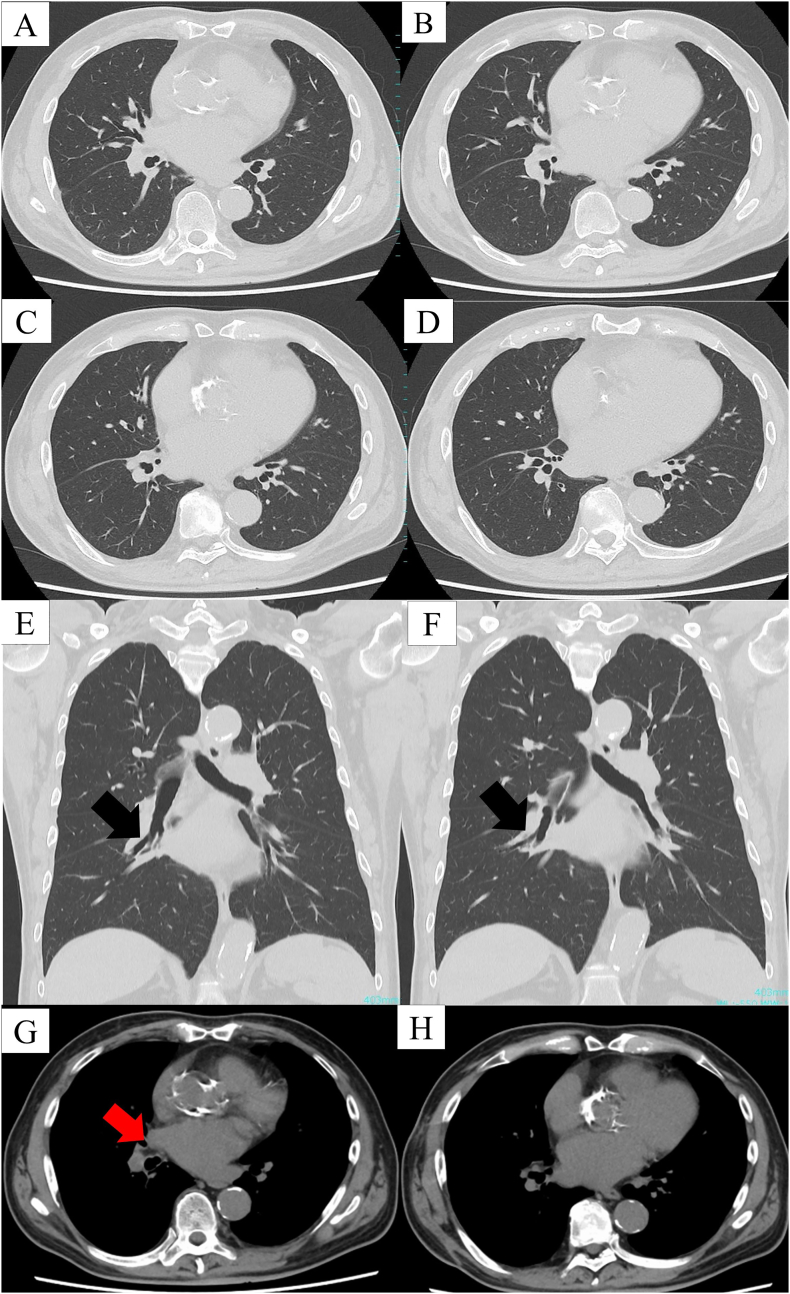
Six-months postoperative computed tomographic (CT) images are shown. (A–H) Six-months postoperative CT demonstrating no evidence of stenosis at the site of the sutured bronchus. The right lung is fully expanded. A black arrow shows the right anterior basal segmental bronchus. A red arrow is indicative of the pericardial flat flap. (For interpretation of the references to colour in this figure legend, the reader is referred to the web version of this article.) (For interpretation of the references to colour in this figure legend, the reader is referred to the web version of this article.)

The postoperative course was uneventful, and the patient was discharged 2 days postoperatively without any complications. He remained reportedly well and asymptomatic when last contacted approximately 1 year postoperatively. Six-months postoperative CT demonstrated that the right lung had fully expanded, with no evidence of a stenosis at the site of the sutured bronchus (Fig. 5A–H).

## 3 Discussion

Despite the excellent results achieved by endoscopic removal [2,[4], [5], [6], [7]], an aspirated foreign body may require surgical intervention [3], particularly in cases of late-diagnosed foreign body aspiration diagnosed ≥1 week post-aspiration [10]. Duan et al. have found that 23 of the 121 late-diagnosed cases (19%), required surgical treatment; however, 81% of foreign bodies were removed using rigid or fiberoptic bronchoscopy [11]. A delayed diagnosis of bronchial foreign bodies is generally related to pulmonary complications, such as pneumonia, atelectasis, and bronchiectasis [12]. Suppurative complications are the most common indications for thoracotomy and pulmonary resection, particularly after the successful endoscopic removal of a foreign body. Wu et al. have observed that only eight of the 3115 patients with bronchial foreign body aspiration underwent open surgery, and all eight patients required pulmonary resection [3]. In patients with bronchial foreign bodies associated with severe respiratory complications, surgical treatment of the damaged lobes must be performed promptly to prevent these complications from progressing into serious lung infections.

Indications for bronchotomy include the failure to remove the foreign body despite repeated attempts, due to immobility, with or without distal bronchial placement. Elaziz et al. have reported on eight cases of thoracotomy and bronchotomy [13]. They have concluded that bronchotomy is a safe and effective alternative to difficult and failed bronchoscopic extractions [13]. In the present case, we performed a bronchotomy, as opposed to a right lower lobectomy, to remove the bronchial foreign body without sacrificing the functioning of the lung parenchyma. Moreover, based on the context of the presence of moderate pulmonary complications, we decided to preserve the right lower lobe, due to the full expansion thereof after the removal of the bronchial foreign body. Postoperative CT confirmed that the right lower lobe had fully expanded.

Furthermore, in the present case, the foreign body was removed via a longitudinal bronchotomy incision of the right basal bronchus, because this incision could potentially be extended if the bronchial foreign body was difficult to remove. Six-months postoperative CT demonstrated no evidence of stenosis at the site of the sutured bronchus. Nevertheless, as per a previous report, a transverse bronchotomy incision may have been less compromising to the lumen after the repair thereof than the longitudinal incision that had been performed [14]. We suggest that the surgical approach regarding bronchotomy incisions should be decided after careful deliberation of the material, shape, size, and location of foreign bodies; as well as the surrounding bronchopulmonary conditions.

Considerable progress has been made regarding VATS. VATS is currently accepted as a reasonable alternative to thoracotomy. To the best of our knowledge, this is the first known case of a successfully performed VATS bronchotomy. Nonetheless, we consider thoracoscopy to be superiorly beneficial in providing better visualization, with an increased likelihood for postoperative recovery, and as a safe and feasible alternative for bronchial foreign body removal.

## 4 Conclusion

Bronchoscopy is the gold standard for foreign body removal; nevertheless, aspirated foreign bodies may require surgical intervention. We presented a case of a bronchial foreign body that was successfully removed by VATS bronchotomy after multiple failed bronchoscopic attempts. Thus, VATS bronchotomy is a viable alternative for foreign body removal.

## Abbreviations

VATS: video-assisted thoracoscopic
CT: Computed tomography

## Consent

Written informed consent was obtained from the patient for publication of this case report and accompanying images. A copy of the written consent is available for review by the Editor-in-Chief of this journal on request.

## Ethical approval

As it is a case report, ethical approval is exempted by University of Miyazaki Hospital.

## Funding

The authors have no competing interests to declare.

## Author contribution

Dr. Fumiya Kawano has designed this report.

Dr. Mayu Inomata and Dr. Ryusei Yamada have reviewed.

Dr. Ryo Maeda is the writer of this article and corresponding author.

## Guarantor

Dr. Ryo Maeda accepts all responsibility of this article.

## Research registration number

Not applicable.

## Conflict of interest statement

All authors have read and approved the final manuscript.
